# Successful conversion surgery for gastric cancer with multiple liver metastases treated after S-1 plus cisplatin combination chemotherapy: a case report

**DOI:** 10.1186/s40792-017-0372-5

**Published:** 2017-08-29

**Authors:** Masashi Tsunematsu, Naoto Takahashi, Keishiro Murakami, Takeyuki Misawa, Tadashi Akiba, Katsuhiko Yanaga

**Affiliations:** 1 Department of Surgery, The Jikei University Kashiwa Hospital, 163-1 Kashiwa-shita, Kashiwa, Chiba Prefecture 277-8567 Japan; 2 Department of Surgery, The Jikei University School of Medicine, 3-19-18 Nishi-shinbashi, Minato-ku, Tokyo, 105-8471 Japan

**Keywords:** Advanced gastric cancer, Multiple liver metastases, Conversion surgery

## Abstract

**Background:**

Gastric cancer with multiple liver metastases have poor prognosis. Recently, stage IV gastric cancer patients who respond well to systemic chemotherapy can be treated by gastrectomy. We herein report a case of advanced gastric cancer with liver metastases who was successfully downstaged by systemic chemotherapy and underwent conversion surgery.

**Case presentation:**

A 60-year-old male patient was diagnosed with gastric cancer with multiple liver metastases [cT3N3M1, stage IV]. After 18 courses of S-1 plus cisplatin combination chemotherapy (S-1 administered orally (80 mg/m^2^/day) twice a day for 21 consecutive days and cisplatin (60 mg/m^2^) infused on day 8), marked regression of liver metastasis was achieved, and we performed open total gastrectomy with D2 lymph node dissection. The patient was discharged from the hospital 10 days after the operation. Histopathological examination revealed no malignant cells in the lymph nodes [ypT1bN0M0, stage IA]. S-1 as the adjuvant chemotherapy was administered for 12 months, and the patient is alive without a recurrence for 33 months after surgery.

**Conclusions:**

Conversion surgery may improve the poor prognosis of gastric cancer.

## Background

Systemic chemotherapy is the standard treatment for stage IV gastric cancer (GC) [1]. During the last decade, several new agents with promising activity against GC have been identified, including S-1, docetaxel, oxaliplatin, and irinotecan [2]. In Japan, S-1 plus cisplatin is currently recognized as a standard treatment for unresectable and metastatic GC with an overall survival (OS) of 13 months in the SPIRITS trial [3].

In the field of colorectal cancer, resection is now actively performed following chemotherapy, particularly in cases of liver metastasis [4]. Conversion surgery is currently recognized as a significant factor for improving life expectancy in cases of advanced and recurrent colorectal cancer.

Conversion surgery for GC is a new issue. It is defined as a surgical treatment aiming at an R0 resection after chemotherapy for tumors that were originally unresectable or marginally resectable for technical and/or oncological reasons [5].

We herein presented a case of conversion surgery for GC with multiple liver metastases treated by S-1 plus cisplatin combination chemotherapy.

## Case presentation

**Fig. 1:**
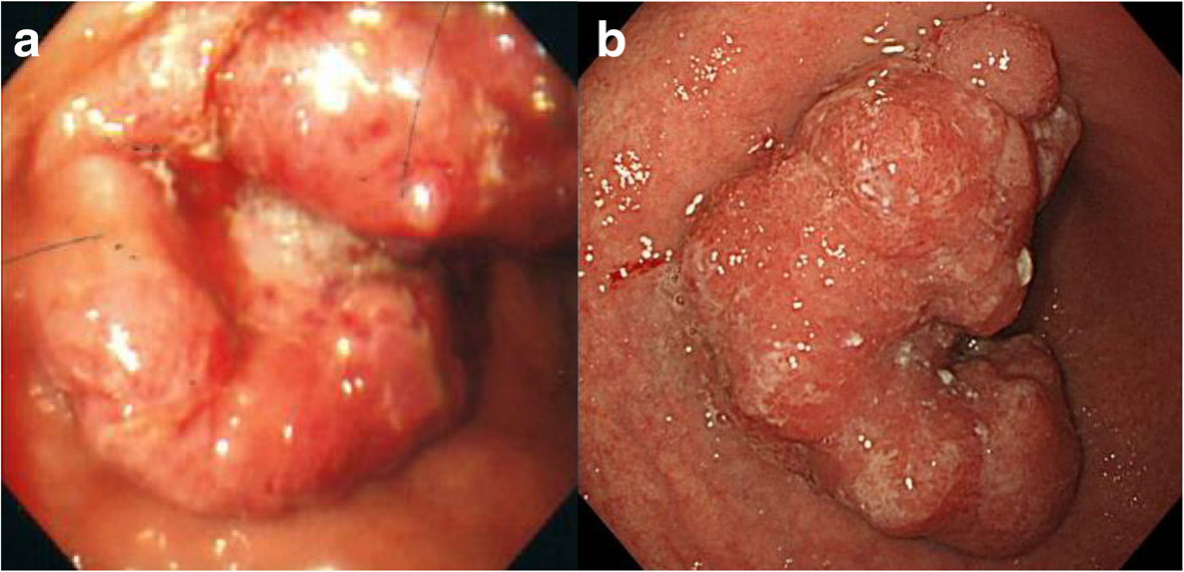
*Uppe*r gastrointestinal endoscopy findings. **a** At the initial visit. Type III advanced GC was identified in the anterior wall of the lower gastric body. **b** Following chemotherapy. A portion of the primary tumor still remained

**Fig. 2:**
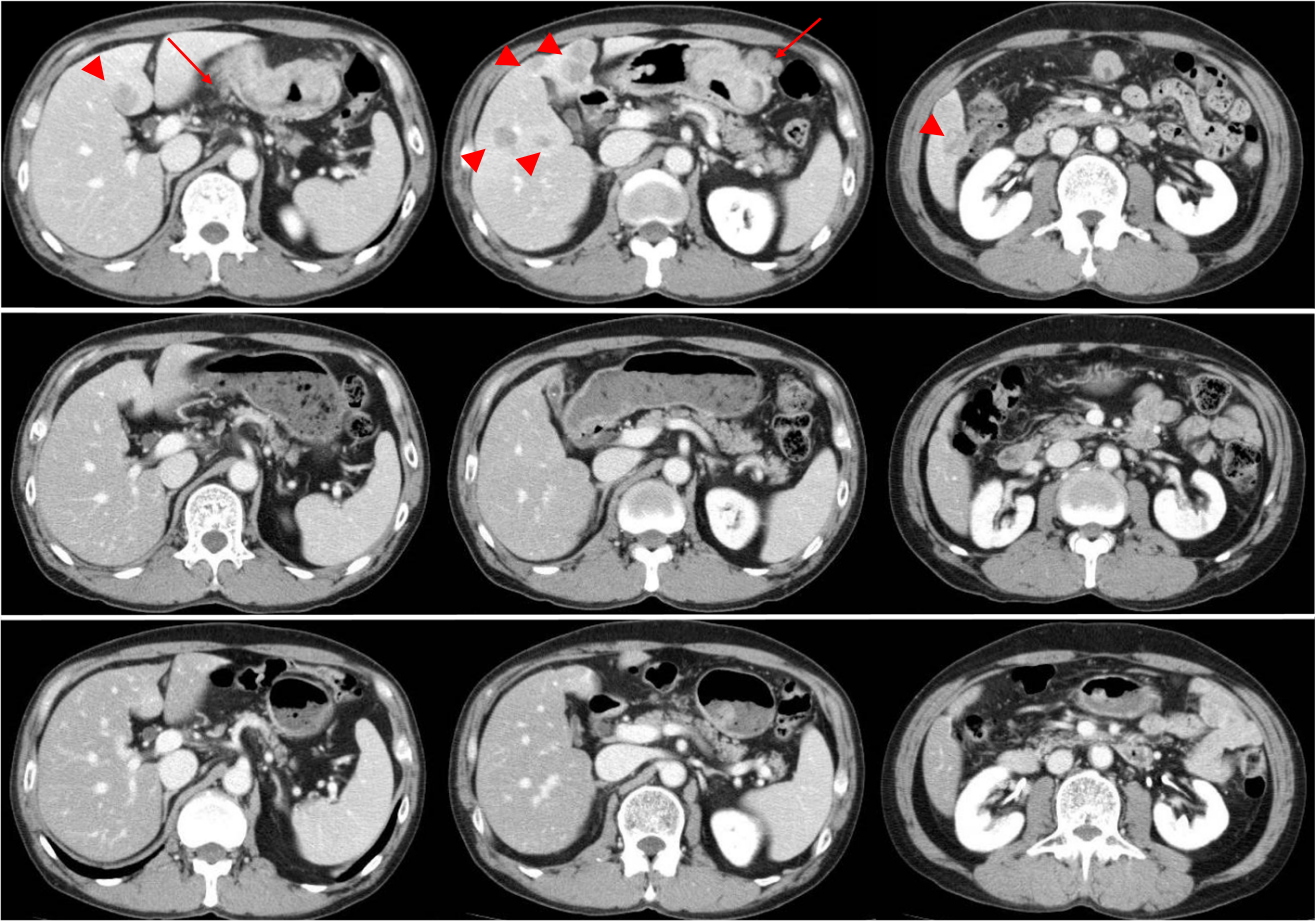
Enhanced computed tomography scan findings. **a** Thickening of the anterior wall in the *lower* gastric body with enlargement of lymph nodes (*arrow*) along with the lesser curvature and greater curvature. Multiple liver metastases (arrowhead). **b**, **c** Complete response of liver metastases and lymph node metastases had been continued

A 60-year-old male (height 162 cm, body weight 50.0 kg, body mass index 19.0) was referred to our hospital presenting with palpitation and dizziness. Upper gastrointestinal endoscopy indicated type III advanced GC in the anterior wall of the lower gastric body (Fig. 1a). Biopsy results yielded a diagnosis of a well-differentiated adenocarcinoma. An enhanced computed tomography scan (eCT) revealed thickening of the anterior wall in the lower gastric body, enlarged lymph nodes along the lesser and greater curvature, and multiple liver metastases (Fig. 2a). The clinical diagnosis was cT3N3M1 stage IV (according to the Japanese classification system), for which systemic chemotherapy (S-1 was administered orally (80 mg/m^2^/day) twice a day for 21 consecutive days, and cisplatin (60 mg/m^2^) was infused on day 8). After six courses of S-1 plus cisplatin combination chemotherapy, the lymph nodes and multiple liver metastases showed remarkable regression without any adverse effects. However, the primary tumor was unaffected (Fig. 2b).

**Fig. 3:**
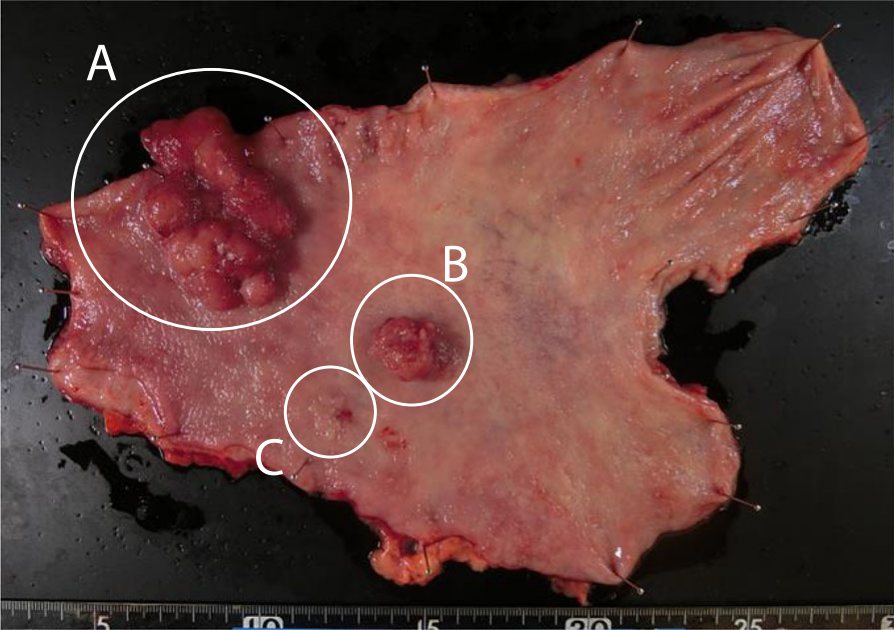
Surgically resected specimen. Tumor in the anterior wall of the gastric body (**a**) with daughter nodules (**b**, **c**)

Since the patient rejected our suggestion for a conversion surgery, 12 more courses of this regimen and 4 courses of S-1 (S-1, 80 mg/m^2^) were administered. The relative dose intensities for S-1 and cisplatin were 48.4 and 74.1%. Adverse effects were grade 2 watering eyes and grade 2 peripheral sensory neuropathy (Common Terminology Criteria for Adverse Events version 4.0). Thereafter, CT demonstrated disappearance of liver metastases and lymph node metastases (Fig. 2c), but upper gastrointestinal endoscopy showed the primary tumor still remained (Fig. 1b). Therefore, after obtaining informed consent, open total gastrectomy with D2 lymph node dissection was performed. The patient was discharged 10 days after surgery without surgical complications. Histopathological specimen revealed malignant cells in the anterior wall of the gastric body (Fig. 3), but no malignant cells in the lymph nodes [ypT1bN0M0, stage IA]. The patient achieved a partial response according to the RECIST 1.1 standard. S-1 was administered as the adjuvant chemotherapy for 12 months, and the patient is alive without a recurrence for 33 months after surgery.

### Discussion

Conversion surgery is an option for unresectable metastatic gastric cancer when distant metastases are controlled by chemotherapy; however, the feasibility and efficacy of conversion surgery for GC remain unclear.

Liver metastasis in GC is frequent, with an incidence of 4–14% [6–9]. Chemotherapy is the main treatment for advanced GC with liver metastasis. New anticancer drugs, such as S-1, capecitabine, paclitaxel, docetaxel, irinotecan, and oxaliplatin have been developed in the last 20 years. Combination treatments using these potent new drugs have been actively introduced in chemotherapy for GC and are contributing to significant improvements in anti-tumor responses and patient survival. However, no prospective trial investigating systemic chemotherapy specified in hepatic metastases has been reported.

On the other hand, the role of surgical removal of liver metastases of GC is still controversial. A small number of patients with limited liver metastasis are thought to gain a survival benefit from hepatectomy, because it usually occurs in the setting of multiple lesions and it recurs at high rate after hepatectomy [10, 11].

These advances in chemotherapy for GC have raised new clinical issues in the treatment of incurable GC patients. During primary chemotherapy, we are presented with an opportunity to manage GC patients in whom factors indicating incurable disease have apparently disappeared or are well controlled by chemotherapy [12]. For such patients, surgery to excise macroscopically remaining disease with curative intent may be an option. This type of surgery for GC, so-called conversion surgery, appears to have potential benefits in terms of patient survival, but it remains unclear whether or not such a conversion surgery can be conducted safely and with certainty and to what extent patient survival is prolonged.

This approach is not equivalent to neoadjuvant chemotherapy, in that neoadjuvant chemotherapy is conducted for the purpose of downstaging only those cancer lesions which are determined clinically resectable from the beginning of chemotherapy. Unfortunately, many surgeons tend to confuse “conversion surgery” with “neoadjuvant chemotherapy”.

We searched cases of conversion surgery for GC with multiple metastases in PubMed using keywords such as “multiple liver metastases,” “gastric cancer,” and “preoperative chemotherapy.” Table 1 shows reports of surgery following chemotherapy for advanced GC with multiple liver metastases [12–18]. These patients were good responders to initial systemic chemotherapy. They achieved a partial response or a complete response of liver metastases with small number of courses. For such patients, conversion surgery is a suitable approach which improves the poor prognosis of stage IV GC. However, there is insufficient evidence regarding the optimal regimen and number of courses required for considering conversion surgery.

**Table 1:** Cases of conversion surgery for GC with multiple metastases

First author	Li ZY	Mitani M	Matsumoto K	Takahashi N	Takahashi N	Kagawa I	Itani Y	Hatta W
Year	2014	2014	2011	2007	2007	2006	2006	2005
Sex	Female	Male	Male	Male	Male	Male	Female	Female
Age (years)	56	75	75	54	57	75	44	73
Location	A	Unknown	L	L	L	M	L	A
Borrmann classification	3	3	3	3	3	3	2	2
Preoperative chemotherapy	Her + OX + S1	S1 + CDDP	S1 + CPT-11	S1 + CDDP	S1 + CDDP	S1 + CDDP	S1 + CDDP	S1
No. of courses	10	4	3	1	1	4	3	1
Operation	DG + PH	DG + RFA	DG	DG	DG	DG + RFA	DG + PH + RFA	DG
Histology differentiation	Poorly differentiated	Poorly differentiated	Tub 1	Tub 1	Poorly differentiated	Tub 1	Tub 2, por 1	Tub 2
Adjuvant chemotherapy	Her + OX + S1	S1	S1 + CPT-11	None	S1 + CDDP	S1	Unknown	None
Recurrence	None	None	None	None	None	None	Unknown	None
Postoperative course	Alive, 3 months	Dead, 7 years	Alive, 3 years	Alive, 12 months	Alive, 17 months	Alive, 15 months	Unknown	Alive, 19 months

*A* antrum, *L* lower gastric body, *Her* trastuzumab, *OX* oxaliplatin, *CDDP* cisplatin, *CPT-11* camptothecin, *DG* distal gastrectomy, *PH* partial hepatectomy, *RFA* radiofrequency ablation

Recently, Yoshida et al. proposed the new biological categories for the classification of stage IV GC [5]. Our case can be classified as category 2 (marginally resectable metastasis). Patients in category 2 would be administered first-line chemotherapy as the induction chemotherapy because it may be able to achieve sufficient response in the areas targeted for resection, and resection of the primary lesion shall be performed after distant metastatic lesions showed clinically complete responses. However, even in such cases, it is difficult to make judgments on whether to continue appropriate chemotherapy or to attempt conversion surgery.

Further studies are needed to evaluate and determine the optimal regimens, as well as the suitable number of courses for each unresectable lesion.

## Conclusions

We reported a case of successful conversion surgery for GC with multiple liver metastases. Conversion surgery may improve the poor prognosis of GC, while further studies and careful assessment are necessary to determine the optimal regimen, as well as the number of courses that will be the ultimate treatment for each case.
